# ZBTB4 Deficiency Exacerbates DSS-Induced Colitis Through Activating NF-κB Pathway

**DOI:** 10.3390/cells15100929

**Published:** 2026-05-18

**Authors:** Xinyi Peng, Genglin Guo, Songyu Li, Songyao Sun, Cong Ouyang, Jiajun Cui

**Affiliations:** 1 The Center for Translational Medicine, Yichun University, Yichun 336000, China; peng_xinyi2001@163.com (X.P.); ggl65316053059@163.com (G.G.); llsy2570@163.com (S.L.); 18815396253@163.com (S.S.); 2 The Department of Biochemistry, School of Basic Medical Sciences, Yichun University, Yichun 336000, China

**Keywords:** inflammation, ulcerative colitis, ZBTB4, Serpine1, NF-κB, handelin

## Abstract

**Highlights:**

**What are the main findings?**

- ZBTB4 deficiency upregulates Serpine1 and then activates NF-κB signaling.
- The natural compound handelin upregulates ZBTB4 and alleviates colitis in a ZBTB4-dependent manner.

**What are the implications of the main findings?**

- ZBTB4 serves as a critical suppressor of ulcerative colitis by restraining NF-κB signaling, highlighting its potential as a therapeutic target.
- Handelin, as a ZBTB4 agonist, may represent a novel and effective approach for treating ulcerative colitis.

**Abstract:**

Inflammatory bowel diseases, particularly ulcerative colitis (UC), are chronic relapsing inflammatory disorders with limited therapeutic options. The zinc-finger transcription factor ZBTB4 has been implicated in the initiation and progression of cancer, but its role in UC remains unknown. Here, we found that *ZBTB4* deficiency exacerbates dextran sulfate sodium (DSS)-induced colitis in C57BL/6J male mice. Compared with the wild type, *ZBTB4* deficiency increases weight loss, colon shortening and proinflammatory cytokine production. RNA-seq analysis revealed that *ZBTB4* deficiency enhances Serpine1 expression and activates the NF-κB pathway. NF-κB inhibition by JSH-23 alleviated the effect of *ZBTB4* deficiency on DSS-induced colitis. These results imply the protective role of ZBTB4 in UC. Through an integrated drug screening, we identified a natural sesquiterpene lactone, handelin, as a potential compound to enhance ZBTB4 expression in NCM460 cells. Handelin administration relieved colitis in wild-type mice but produced no effect in *ZBTB4* knockout mice, demonstrating that its anti-colitic effect depends on ZBTB4 expression. Collectively, our results indicate the key role of ZBTB4 in UC and ZBTB4 agonists may serve as a novel approach for UC treatments.

## 1. Introduction

Inflammatory bowel diseases (IBDs), including the chronic, relapsing inflammatory conditions Crohn’s disease and ulcerative colitis (UC), represent a growing global health burden due to their steadily increasing incidence [1,2]. Ulcerative colitis, characterized by inappropriate and sustained activation of the mucosal immune system, is triggered by commensal intestinal microbiota in the context of impaired intestinal epithelial barrier function and dysregulated mucosal immunity [3]. Current therapeutic strategies for UC primarily aim to offer symptomatic relief and improve the life quality of patients [4]. The mainstay pharmacological agents include aminosalicylates, glucocorticoids, immunomodulators, biologics, and small-molecule drugs [5]. However, these treatment modalities are often limited by high relapse rates and a range of adverse effects [6].

The Zinc Finger and Broad-Complex, Tramtrack, and Bric-à-Brac (BTB) domain-containing (ZBTB) family comprises approximately 50 members and constitutes one of the largest families of transcription factors in humans [7,8]. Several ZBTB proteins have been implicated in lymphocyte development, and accumulating evidence indicates that multiple members of this family play critical roles in immune regulation [9]. Notably, certain ZBTB proteins—including Kaiso, HIC1, and Th-POK—have been shown to exert functional effects in the intestinal tract [10,11,12]. ZBTB4, another member of this family, has been primarily associated with various types of cancers [13]. Intriguingly, in systemic juvenile idiopathic arthritis (sJIA), the long non-coding RNA MALAT1 modulates the JAK/STAT signaling pathway through the miR-150-5p/ZBTB4 axis, thereby influencing the production of proinflammatory cytokines [14]. This study suggests that ZBTB4 may serve as a pivotal regulator in inflammatory processes. However, the role of ZBTB4 in ulcerative colitis remains unclear.

In this study, we aimed to investigate the regulatory role of ZBTB4 in ulcerative colitis. We showed that *ZBTB4* deficiency exacerbates DSS-induced colitis in mice, accompanied by increased proinflammatory cytokine production and colonic damage. Mechanistically, ZBTB4 loss upregulates Serpine1 and hyperactivates the NF-κB signaling pathway. Pharmacological inhibition of NF-κB rescues the severe colitis phenotype in *ZBTB4*-deficient mice. Furthermore, we identified handelin as a natural inducer of ZBTB4 expression. Handelin alleviates colitis in wild-type mice but fails to protect *ZBTB4* knockout mice, indicating that its anti-colitic effect depends on ZBTB4. In conclusion, our study defines ZBTB4 as a critical suppressor of ulcerative colitis through restraining NF-κB signaling and highlights handelin as a potential therapeutic agent acting via ZBTB4 upregulation.

In this study, we observed that ZBTB4 is an important regulator for DSS-induced colitis in mice. *ZBTB4* deficiency exacerbates DSS-induced colitis through activating the NF-κB signaling pathway. A natural sesquiterpene lactone, handelin, can induce ZBTB4 expression and relieve colitis in mice.

## 2. Materials and Methods

### 2.1. Cell Culture and Reagents

Human colonic epithelial NCM460 cell line (The Chinese Academy of Sciences, Shanghai, China) was authenticated using STR profiling. Cells were maintained in DMEM (Dulbecco’s modified Eagle’s medium) with 10% FBS (fetal bovine serum) in a cell culture incubator. DMEM was obtained from Hyclone (Logan, UT, USA). FBS was obtained from Gibco (Grand Island, NY, USA). Dextran sulfate sodium salt (DSS; molecular weight 36,000–50,000 Da) was purchased from MP Biomedicals (Santa Ana, CA, USA). ELISA kits for the detection of TNF-α, IL-1β, and IL-6 were obtained from 4A Biotech Co., Ltd. (Beijing, China). JSH-23, an inhibitor of NF-κB, was acquired from MedChemExpress (Monmouth Junction, NJ, USA).

### 2.2. Animals

This protocol was approved by the Ethical Committee of the Yichun University of Medical School under approval number 2023(040). The Animal Care and Use Protocol complied with the ARRIVE guidelines and the U.K. Animal Act, 1986, and the associated guidelines, EU Directive 2010/63/EU for animal experiments. Heterozygous ZBTB4 knockout (*ZBTB4^+/−^)* mice were generated by Cyagen Biosciences (Suzhou, China). *ZBTB4^−/−^* mice were generated by intercrossing heterozygous breeders and genotyping was performed to confirm the genotype. Research staff had received special training in animal care and handling. The animals were identified by numbered cages and ear punches. Each cage had a maximum of four mice, and cage positions were randomly arranged and regularly rotated to avoid location bias. All mice were housed under specific pathogen-free (SPF) conditions in a climate- and light-controlled facility at 23 ± 1 °C with a 12 h light/dark cycle. All mice were housed in a quiet environment, and all mice had ad libitum access to sterile water and standard rodent chow. Animals were allocated by age, body weight, and genotype to balance baseline conditions across groups. Seven- to eight-week-old male mice (male mice were used to avoid the interference of estrogen) with comparable body weights (20–22 g) were randomly assigned to experimental groups in all experiments [15]. Mice were monitored daily for health issues such as weight loss, loss of mobility etc. DSS-treated mice were immediately euthanized by gradually increasing the concentration of CO_2_ in the anesthesia chamber if they reached more than 20% body weight loss. All efforts were made to minimize animal discomfort and potential confounders in the mouse experiment.

A double-blind design was employed in all experiments. Principal investigator Jiajun Cui was aware of the group allocation at the different stages of mouse experiments. Other investigators, including Xinyi Peng, were blinded to mouse group assignments during all stages of experimentation, animal handling, and data analysis.

The genotyping primers used in this study are listed as follows:
Forward primer-1: 5′-CTTGATTTCTGACACAGTTTCCGTT-3′.
Reverse primer-1:5′-ATGAAAGAAACAAGGACAGGGAAAC-3′.
Forward primer-2: 5′-GTCTTTGGCTACGCAGTGAATC-3′.
Reverse primer-2:5′-AAGTCTAGGAGCACAGGAAGGAAT-3′.

### 2.3. DSS-Induced Acute Colitis and Treatment

Although the DSS-induced acute colitis model does not fully recapitulate the chronic, recurrent, and multifactorial pathology of human inflammatory bowel disease (IBD), it remains widely used due to its high consistency with the clinical features of human ulcerative colitis. This chemically induced model primarily manifests acute intestinal epithelial injury and innate immune responses [16]. Colitis was induced by administering DSS (36,000–50,000 MW, MP Biomedicals) in drinking water (2.5%, *w*/*v*) for seven consecutive days, while control mice received sterile ddH_2_O. Certain imprecision may exist in the results due to individual biological differences among mice, slight variations in DSS potency between different batches, and fluctuations in daily water intake, all of which may lead to minor differences in disease phenotype.

JSH-23 was dissolved in corn oil (10 mg/mL stock), diluted to 0.3 mg/mL working solution, and then administered by oral gavage at 10 mL/kg (3 mg/kg) once daily in the morning according to previous studies [17,18]. Handelin was dispersed in 0.5% CMC-Na to a final concentration of 2 mg/mL (12 mg in 6 mL) and administered by oral gavage at 10 mL/kg (20 mg/kg) once daily in the morning according to previous studies [19].

### 2.4. Disease Activity Index (DAI) Evaluation

Mice were monitored daily throughout the experimental period for general health, activity, food and water intake, body weight changes, stool consistency, and fecal occult blood. The disease activity index (DAI) was calculated as the sum of scores for three parameters: (1) weight loss (%), (2) stool consistency, (3) presence of fecal blood, as previously described.

### 2.5. ELISA

Colonic tissues were homogenized in ice-cold PBS using a glass homogenizer. Homogenates were centrifuged at 12,000× *g* for 15 min at 4 °C, and supernatants were collected. Total protein concentrations were determined using a BCA protein assay kit obtained from Thermo Fisher Scientific (Waltham, MA, USA). Levels of TNF-α, IL-1β, and IL-6 in tissue lysates were quantified using commercial ELISA kits (4A Biotech, Beijing, China) according to the manufacturer’s instructions. Absorbance was measured at 450 nm using a microplate reader of Multiskan FC obtained from Thermo Fisher Scientific (Waltham, MA, USA).

### 2.6. Western Blot Analysis

Total protein was extracted from colonic tissues or cultured cells using RIPA buffer containing PMSF, protease inhibitors, and phosphatase inhibitors. After centrifugation (12,000× *g*, 15 min, 4 °C), supernatants were collected, and protein concentrations were determined by BCA assay. Equal amounts of protein were mixed with 4× SDS loading buffer, denatured at 95 °C for 5 min, and resolved by SDS-PAGE on 8–12% polyacrylamide gels. Proteins were then transferred onto nitrocellulose membranes obtained from Bio-Rad Laboratories (Hercules, CA, USA). Membranes were blocked with 5% non-fat skim milk in Tris-buffered saline containing 0.1% Tween-20 (TBST) for 1 h at room temperature and subsequently incubated overnight at 4 °C with primary antibodies. After washing, membranes were incubated with appropriate horseradish peroxidase (HRP)-conjugated secondary antibodies. Protein bands were visualized using the ECL Plus Western Blot Detection System (Cytiva) (Washington, DC, USA) and imaged with a chemiluminescence detection system.

### 2.7. Histological Analysis (H&E Staining)

Colon segments were fixed in 10% neutral buffered formalin, embedded in paraffin, and sectioned at 5 μm thickness. Sections were stained with hematoxylin and eosin (H&E) and scanned using a NanoZoomer 2.0 RS slide scanner obtained from Hamamatsu Photonics (Shizuoka, Japan).

### 2.8. Quantitative Real-Time PCR (qRT-PCR)

Total RNA was isolated from colonic tissues or cells using TRIzol Reagent obtained from Invitrogen (Waltham, MA, USA). First-strand cDNA was synthesized from 1 μg of total RNA using the High-Capacity cDNA Reverse Transcription Kit obtained from Applied Biosystems (Waltham, MA, USA). Gene expression levels of *ZBTB4* and the housekeeping gene *GAPDH* were quantified using SYBR Green Master Mix (Applied Biosystems) on a StepOnePlus™ Real-Time PCR System (Applied Biosystems). Relative mRNA expression was calculated using the 2^−ΔΔCt^ method, with Gapdh as the internal reference. The primers used in qRT-PCR are listed as follows:

ZBTB4 forward primer: 5′-CCATCGCAGTCAGAAGGAAGA-3′.
ZBTB4 reverse primer:5′-GTGAGCAGGGAACTTGGTGT-3′.
GAPDH forward primer: 5′-AGGTCGGAGTCAACGGATTT-3′.
GAPDH reverse primer: 5′-TTCCCGTTCTCAGCCTTGAC-3′.

### 2.9. RNA Sequencing (RNA-Seq)

Samples were immediately snap-frozen in liquid nitrogen and stored at −80 °C until processing. Total RNA was extracted, and transcriptome sequencing was performed by a certified genomics service provider. RNA integrity, library preparation, and sequencing depth followed standard quality control protocols. Gene set enrichment analysis (GSEA) was plotted by https://www.bioinformatics.com.cn (last accessed on 1 April 2025), an online platform for data analysis and visualization.

### 2.10. Statistical Analysis

Sample size was determined based on previous studies using the DSS-induced colitis mouse model and our preliminary experiment [15]. No statistical method was used to predetermine the sample size. We used 10–15 mice per group to ensure sufficient statistical power and reliable results. GraphPad prism 7.0 was used in statistical analysis and plotting. Unless specified, three independent experiments were conducted and data are presented as mean ± SEM. For group comparisons, Student’s t-test and one-way or two-way ANOVA were used. *p* < 0.05 was set as statistically significant.

## 3. Results

### 3.1. ZBTB4 Deficiency Exacerbates DSS-Induced Colitis

**Figure 1:**
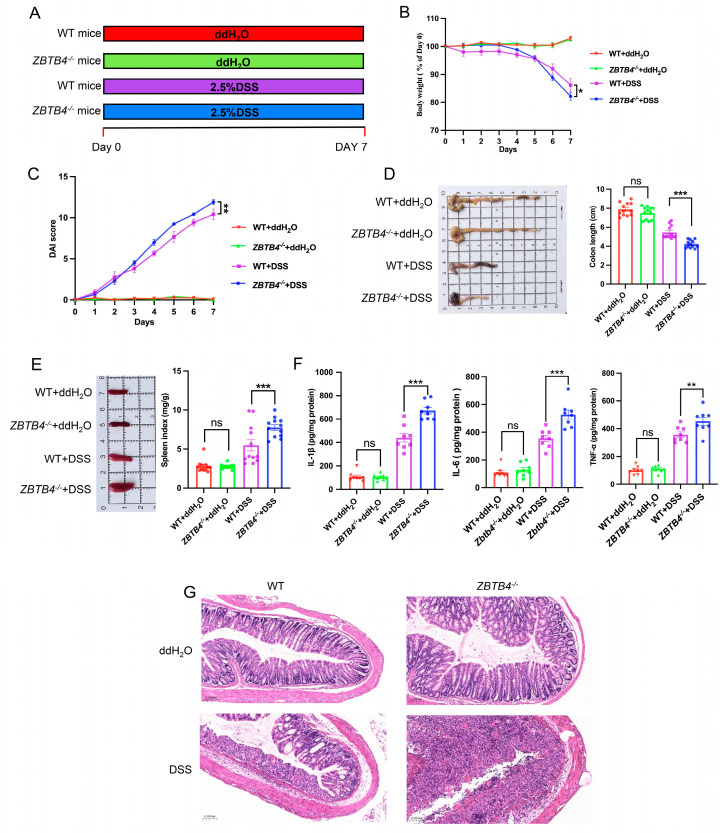
ZBTB4 deficiency exacerbates DSS-induced colitis. A total of 60 mice were used, with 15 mice allocated to each experimental group. One mouse in the WT + DSS group and three mice in the *ZBTB4*^−/−^ + DSS group died during the experiment and were excluded from subsequent data analysis. All exclusions were due to mortality related to severe DSS-induced colitis or other health issues. (**A**) Schematic illustration of the DSS-induced colitis protocol. (**B**,**C**) The degree of body weight loss and DAI scores of each group of mice were measured. (**D**,**E**) The gross morphology images of colons and spleens were assessed and colon lengths and the spleen index in WT and *ZBTB4*^−^/^−^ mice were measured (n = 12 per group, four groups). (**F**) Concentrations of TNF-α, IL-1β, and IL-6 were quantified by ELISA in colon homogenates from WT and *ZBTB4*^−/−^ mice (n = 8 per group, four groups; 4 of the 12 mice per group were not included in the analysis, as they were randomly assigned to the preliminary experiment). (**G**) Colonic sections of each group of mice were stained by H&E. **: *p* < 0.01, ***: *p* < 0.001, ns: no significance.

To investigate the role of ZBTB4 in colitis, we successfully generated *ZBTB4* knockout (*ZBTB4*^−/−^) mice (Figure S1). Then, colitis was induced by administering 2.5% dextran sulfate sodium (DSS) in drinking water for seven consecutive days, and the control group were fed with sterile ddH_2_O as shown in Figure 1A. As a result, DSS-treated mice exhibited the typical phenotype of UC such as body weight loss, colon shortening, increased spleen index, elevated disease activity index (DAI), and severe mucosal injury. Compared to the wild-type (WT) mice, *ZBTB4*^−/−^ mice exhibited more severe colitis with increased weight loss and colon shortening, along with an elevated DAI score and spleen index (Figure 1B–E).

Ulcerative colitis is closely associated with dysregulated inflammation and is characterized by excessive production of proinflammatory cytokines including tumor necrosis factor-alpha (TNF-α), interleukin-1 beta (IL-1β), and interleukin-6 (IL-6) [20]. To assess the inflammatory status at the molecular level, we measured cytokine concentrations in colonic tissues by enzyme-linked immunosorbent assay (ELISA). We found that *ZBTB4*^−/−^ mice with colitis exhibited increased levels of TNF-α, IL-1β, and IL-6 (Figure 1F). We also detected ZBTB4 protein expression in colon tissues from WT and DSS-treated mice. The results showed that ZBTB4 expression had no significant difference between the two groups (Figure S2A,B). Furthermore, hematoxylin and eosin (H&E) staining of colonic sections demonstrated that *ZBTB4*^−/−^ mice exhibited more severe disruption of intestinal architecture and extensive inflammatory cell infiltration than the wild-type mice (Figure 1G). Collectively, these results indicate that *ZBTB4* deficiency exacerbates DSS-induced colitis.

### 3.2. ZBTB4 Deficiency Increases Colonic Serpine1 Expression and Activates NF-κB Pathway in DSS-Induced Colitis Mice

**Figure 2:**
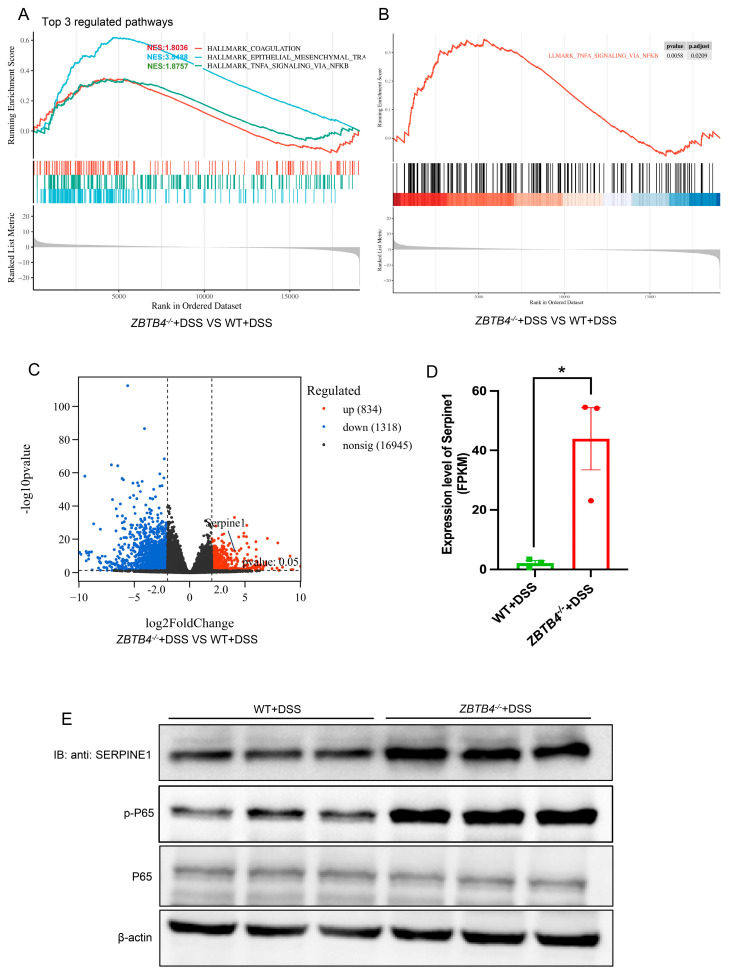
ZBTB4 deficiency increases colonic Serpine1 expression and activates NF-κB pathway in DSS-treated mice. (**A**) Top 3 pathways differentially enriched in colonic tissues of DSS-treated WT versus *ZBTB4*^−/−^ mice, as determined by gene set enrichment analysis (GSEA) of RNA sequencing data (n = 3 per group, two groups). Genes were ranked by signal-to-noise ratio; significance was defined as q value < 0.05 and |NES| > 1.8. (**B**) GSEA revealed significant enrichment of the Hallmark “TNFA_SIGNALING_VIA_NFKB” gene set in *ZBTB4*^−/−^ colitis mice (NES = 1.88, q value < 0.01). (**C**) Volcano plot showing the expression of cytokines, chemokines, and key components of the NF-κB signaling pathway in colonic tissues from WT and *ZBTB4*^−/−^ mice with DSS-induced colitis. (**D**) Serpine1 expressions in colonic tissues from DSS-induced colitic WT and *ZBTB4*^−/−^ mice were determined by RNA-seq. (**E**) Protein levels of Serpine1, p65 (RELA), and phosphorylated p65 in colonic tissues from WT and *ZBTB*4^−/−^ mice which were treated with DSS were assessed by Western blot. *: *p* < 0.05.

To investigate the mechanisms underlying the role of ZBTB4 in colitis, we performed RNA sequencing (RNA-seq) using colonic tissues in the WT and *ZBTB4*^−/−^ mice which were treated with DDS. Gene set enrichment analysis (GSEA) of the RNA-seq data revealed a significant enrichment of the NF-κB signaling pathway in *ZBTB4*^−/−^ mice compared to the WT (Figure 2A,B). Comparative transcriptomic analysis identified 2152 differentially expressed genes (DEGs) out of 19,097 detected transcripts between the two groups. Among these genes, we found that 834 genes were upregulated and 1318 were downregulated in *ZBTB4*^−/−^ mice (defined as |log_2_ fold change| ≥ 2 and *p* < 0.05) (Figure 2C). Of note, *Serpine1* was more than 40-fold higher in *ZBTB4*^−/−^ mice than the WT (Figure 2D). Serpine1 has been shown to be the key activator of the NF-κB signaling pathway through promoting the nucleolar translocation of p65 (RelA), a core subunit of the NF-κB transcription factor complex [21]. Therefore, we decided to carry out experiments to study the role of Serpine1 and NF-κB in the protective effect of ZBTB4 on DSS-induced colitis. Our Western blot analysis confirmed the upregulation of Serpine1 in colonic tissues of *ZBTB4*^−/−^ mice. More importantly, we observed the increased phosphorylation of p65, the active form of the NF-κB complex (Figure 2E). Collectively, these results indicate that *ZBTB4* deficiency led to Serpine1 upregulation and activation of the NF-κB signaling cascade during DSS-induced colitis.

### 3.3. ZBTB4 Deficiency Exacerbates Ulcerative Colitis by Activating the NF-κB Pathway

**Figure 3:**
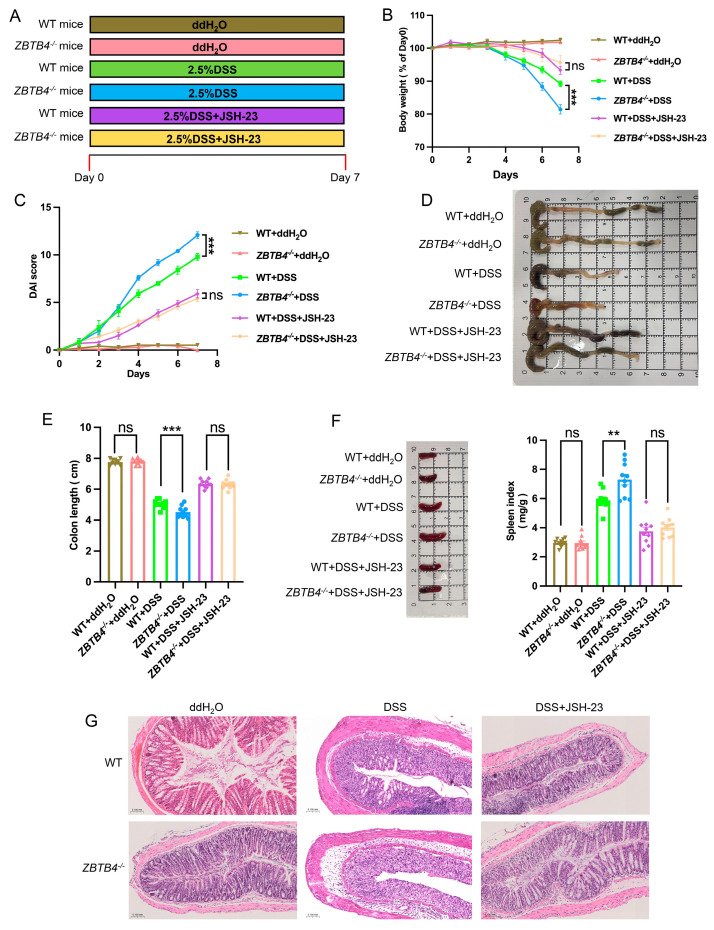
ZBTB4 deficiency exacerbates ulcerative colitis by activating the NF-κB pathway. A total of 72 mice were used, with 12 mice allocated to each experimental group. One mouse in the *ZBTB4*^−/−^ + DSS group died during the experiment and was excluded from subsequent data analysis. All exclusions were due to mortality related to severe DSS-induced colitis or body weight reduction of more than 20% or other health issues. (**A**) Schematic illustration of the experimental design for DSS-induced colitis and NF-κB inhibition with JSH-23. (**B**,**C**) The degree of body weight loss and DAI scores of each group of mice were measured (n = 10 per group, six groups). (**D**–**F**) The gross morphology images of colons and spleens and colon lengths and spleen index in mice as indicated in the absence or presence of JSH-23 (n = 10 per group, six groups). (**G**) Colonic sections of each group of mice were stained by H&E. **: *p* < 0.01, ***: *p* < 0.001, ns: no significance.

Our data above suggested that *ZBTB4* deficiency enhanced the activity of NF-κB. Then, we decided to carry out experiments to examine if NF-κB activation mediates the exacerbating effect of *ZBTB4* deficiency on ulcerative colitis. To address this possibility, we treated mice with JSH-23, a specific NF-κB inhibitor, along with DSS as shown in Figure 3A. As a result, JSH-23 treatment significantly reversed the exacerbating effect of *ZBTB4* deficiency including body weight and colon length and decreased the disease activity index (DAI) scores and spleen index in *ZBTB4*^−/−^ mice (Figure 3B–F). Furthermore, Serpine1 expression in colonic tissues from different groups was consistent with the severity of DSS-induced colitis, indicating that ZBTB4 deficiency leads to Serpine1 upregulation and activation of the NF-κB signaling cascade during DSS-induced colitis (Figure S3A,B). Hematoxylin and eosin (H&E) staining of colonic sections also indicated the diminishing effect of JSH-23 on *ZBTB4* deficiency-induced colitis (Figure 3G). Taken together, these findings suggested that NF-κB activation mediates the exacerbating effect of *ZBTB4* deficiency on ulcerative colitis.

### 3.4. Handelin Alleviates Ulcerative Colitis in a ZBTB4-Dependent Manner

**Figure 4:**
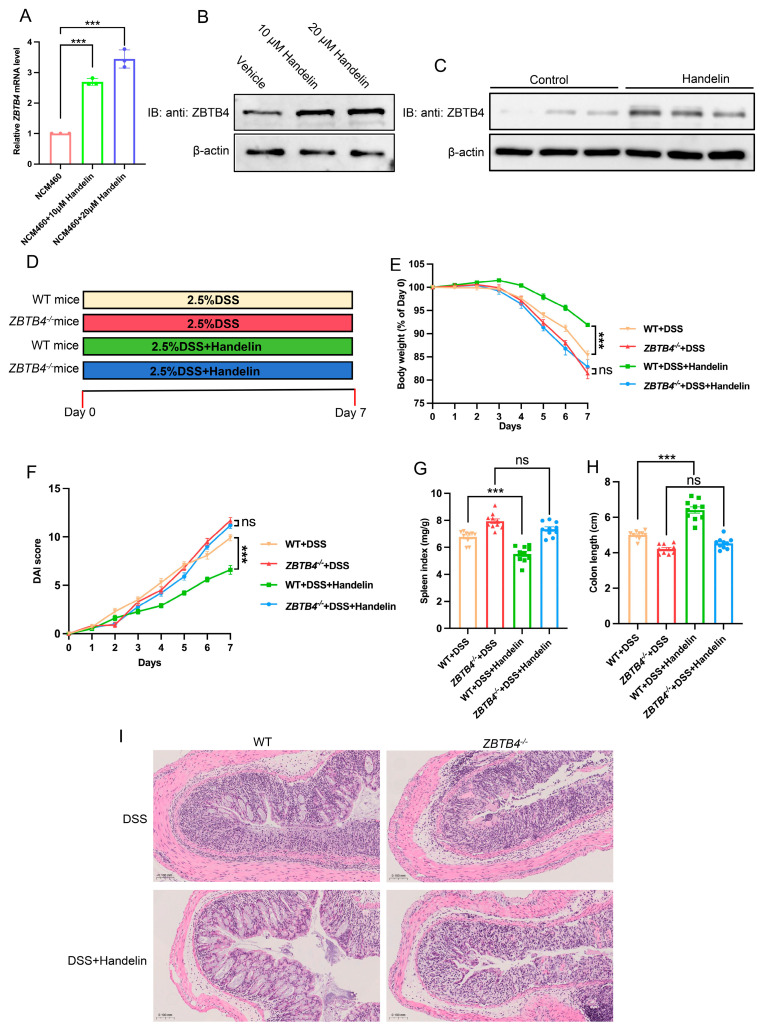
Handelin alleviates ulcerative colitis in a ZBTB4-dependent manner. A total of 48 mice were used, with 12 mice allocated to each experimental group. All exclusions were due to a body weight reduction of more than 20% or other health issues. (**A**,**B**) NCM460 cells were treated with handelin (10 μM and 20 μM) for 24 h. ZBTB4 mRNA and protein levels were measured by RT-qPCR and Western blot, respectively. (**C**) WT and *ZBTB4*^−/−^ mice were treated with handelin for 7 days. ZBTB4 protein levels were measured by Western blotting. (**D**) Schematic illustration of the experimental design for DSS-induced colitis and the treatment with handelin. (**E**–**H**) The degree of body weight loss and DAI scores of each group mice were measured. The colon lengths and spleen index in each group of mice as indicated were measured (n = 10 per group, four groups). (**I**) Colonic sections of each group of mice were stained by H&E. ***: *p* < 0.001, ns: no significance.

Our data above demonstrated that *ZBTB4* deficiency exacerbates ulcerative colitis in mice. These findings indicate that ZBTB4 could protect mice against DSS-induced colitis and we deduced that upregulation of ZBTB4 might alleviate ulcerative colitis. Thus, we next sought to identify pharmacological agents which are able to enhance ZBTB4 expression. We performed an integrated drug screening using the Comparative Toxicogenomics Database (CTD), the Traditional Chinese Medicine Systems Pharmacology Database (TCMSP), and the MedChemExpress (MCE) compound library to identify natural compounds which are linked to ZBTB4 expression, autoimmune disorders, and the NF-κB signaling pathway as shown in Figure S2A. The candidate compounds were used to treat NCM460 cells and then ZBTB4 expressions were measured by RT-qPCR. We found that handelin treatment led to the highest level of ZBTB4 expression (Figure S2B and Figure 4A). Western blot analysis also identified handelin as the potential compound to activate ZBTB4 expression (Figure 4B). To validate the in vivo efficacy of handelin, mice were orally administered vehicle or handelin for seven days. Then, colonic tissues were harvested and ZBTB4 expression was measured by Western blot. Handelin-treated mice exhibited a significant increase in ZBTB4 expression compared to the vehicle group (Figure 4C). Given this observation, we hypothesized that handelin may ameliorate colitis in a ZBTB4-dependent manner. To address this possibility, we treated WT and *ZBTB4*^−/−^ mice with handelin or vehicle, along with DSS (Figure 4D). Body weight and DAI score were monitored daily. As a result, handelin treatment markedly alleviated DSS-induced body weight loss and DAI alteration in WT mice but produced no effect in *ZBTB4*^−/−^ mice (Figure 4E,F). After mice were sacrificed, we found that handelin significantly alleviated colon shortening and splenomegaly in WT but not *ZBTB4*^−/−^ mice (Figure 4G,H). In addition, H&E staining further confirmed the anti-colitis effect of handelin in WT but not *ZBTB4*^−/−^ mice. Collectively, these results indicated that the anti-colitic effect of handelin is dependent on ZBTB4.

## 4. Discussion

Through this work, we defined the key role of ZBTB4 in UC and activating ZBTB4 expression might be an alternative strategy for UC treatments. First, we showed that *ZBTB4* knockout (*ZBTB4*^−^/^−^) mice exhibit exacerbating colitis induced by DSS. Then, RNA-seq analysis suggested that *ZBTB4* deficiency upregulates the expression of Serpine1 and then activates NF-κB pathway in DSS-treated mice. Third, we found that *ZBTB4* deficiency exacerbates ulcerative colitis by activating the NF-κB pathway. Finally, a nature compound, handelin, enhances ZBTB4 expression and alleviates ulcerative colitis in a ZBTB4-dependent manner.

UC is widely recognized as a progressive disease driven by persistent inflammation and impaired epithelial barrier integrity [22,23,24]. Dysregulated immune responses are central to mucosal damage, making therapeutic strategies that simultaneously suppress inflammatory cell activation and reinforce epithelial barrier function particularly promising [25,26]. ZBTB proteins belong to zinc-finger proteins (ZFPs) and the BTB domain-containing protein family. ZBTB proteins are primarily considered critical determinants of T cell development and lymphopoiesis. Although the associations of ZBTB proteins with immune infiltration are well defined, their roles in immune diseases are less understood [27,28,29]. A recent report suggests ZBTB20 activation contributes to early-stage osteoarthritis [30]. Another ZBTB protein, ZBTB33, has been shown to regulate the severity of DSS-induced colitis [31]. ZBTB4, the closet homolog member of ZBTB33, has been fully illustrated to be a tumor repressor in various cancers. As a sharp contrast, the role of ZBTB4 in immune disorders is poorly understood [32,33]. Here, we generated *ZBTB4* knockout mice to study the role of ZBTB4 in UC. As a result, we observed that *ZBTB4*^−/−^ mice exhibited significantly more severe manifestations of DSS-induced colitis compared to wild-type controls. This result implies the protective effect of ZBTB4 against DSS-induced colitis.

During UC flares, proinflammatory cytokines, such as TNF-α, IL-1β, and IL-6, are overproduced, contributing to hallmark clinical and histopathological features. The NF-κB pathway is a well-established driver for the production of these cytokines [34,35]. Our RNA-seq analysis implicates Serpine1, a serine protease inhibitor and established mediator of inflammation, as a key downstream effector of ZBTB4. It has been shown that elevated Serpine1 expression, implicated in multiple inflammatory disorders including UC, promotes p65 nuclear translocation to amplify NF-κB signaling and exacerbate inflammatory responses [21,36]. Gene set enrichment analysis (GSEA) demonstrated enhanced NF-κB signaling activity in the colons of DSS-treated *ZBTB4*^−/−^ mice. The NF-κB complex comprises multiple subunits—including p50, p52, p65 (RelA), c-Rel, and RelB. Phosphorylated P65 is a marker of activation for the NF-κB inflammatory pathway. Notably, Serpine1 has been shown to promote p65 nuclear translocation, thereby activating NF-κB signaling [37,38]. In line with this, we observed elevated protein levels of Serpine1 and phosphorylated p65 in the colons of *ZBTB4*-deficient mice. To confirm whether this enhanced NF-κB activity mediates the severe colitis phenotype, we treated DSS-induced colitis mice with JSH-23, an inhibitor of p65. JSH-23 treatment diminished the alleviating effect of *ZBTB4* deficiency on DSS-induced colitis. These data collectively demonstrate that the exacerbated colitis phenotype in *ZBTB4*^−/−^ mice is mediated primarily through activating NF-κB.

Chronic intestinal inflammation is a well-established driver of colitis-associated colorectal cancer (CAC) [39,40]. Since ZBTB4 deficiency hyperactivates NF-κB signaling—a key mediator linking inflammation and tumorigenesis—ZBTB4 likely serves a dual role in both inflammation and tumorigenesis. As a tumor suppressor, ZBTB4 maintains genomic stability and inhibits oncogenic proliferation [41]. We deduced that ZBTB4 may serve as a bridge to link chronic colitis and colitis-associated colorectal cancer. It is interesting to study the relationship between DSS-induced colitis and colorectal cancer initiation using this animal model. Thus, enhancing ZBTB4 expression may not only alleviate colitis but also reduce the risk of CAC in the long term. Thus, we next explored natural compounds capable of enhancing its expression. In human colonic epithelial NCM460 cells, handelin was observed to significantly increase ZBTB4 expression at both mRNA and protein levels. Handelin, a bioactive sesquiterpene lactone derived from Chrysanthemum species, is a traditional Chinese herbal medicine historically used for treating COPD, muscle atrophy and inflammation and this compound produces low toxicity in the human body [19,42,43,44]. We confirmed the effect of handelin on ZBTB4 expression by oral administration of handelin in mice. Importantly, we found that handelin treatment markedly improved clinical and histological outcomes in WT but not in *ZBTB4*^−/−^ mice. It will be interesting to study the effect of handelin in ZBTB4 low-expression and high-expression groups of mice in a future study. For the possible dual roles of ZBTB4 in colitis and CAC, it is worthwhile studying the effects of handelin on the initiation and progression of both colitis and CRC in a future study.

## Data Availability

The original contributions presented in this study are included in the article/Supplementary Materials. Further inquiries can be directed to the corresponding authors.

## Supplementary Materials

The following supporting information can be downloaded at: https://www.mdpi.com/article/10.3390/cells15100929/s1, Figure S1. Construction strategy and identification of *ZBTB4*^−/−^ mice. (A) Illustration of the *ZBTB4* knockout mouse-generating protocol. (B) Sequencing validation of the Zbtb4 knockout allele. (C) Genotyping was performed using two primer pairs: F1/R1 and F2/R2. F1/R1 produces a fragment only present in *ZBTB4*^−/−^, whereas F2/R2 produces a fragment only present in the wild-type allele. (D) The expression of ZBTB4 in WT mice and *ZBTB4*^−/−^ mice was measured by Western blot. Figure S2. ZBTB4 expression in colon tissues from wild-type mice. (A,B) Protein levels of ZBTB4 in colonic tissues from WT mice with or without DSS-induced colitis were assessed by Western blot and quantitatively analyzed with three biological replicates for ZBTB4 expression by ImageJ software (Fiji for Mac OS X). Data are presented as mean ± SEM. Figure S3. ZBTB4 deficiency increases colonic Serpine1 expression. (A,B) Protein levels of Serpine1 in colonic tissues from six groups of mice as indicated were assessed by Western blot and quantitatively analyzed with three biological replicates for Serpine1 expression by ImageJ software. Data are presented as mean ± SEM. *: *p* < 0.05, ns: no significance. Figure S4. Candidate compound screening in vitro. (A) The screening process of candidate compounds to regulate ZBTB4 expression. (B) NCM460 cells were treated with compounds as indicated for 24 h. Three independent experiments were conducted and data are presented as mean ± SD. ZBTB4 mRNA levels were quantified by RT-qPCR. *: *p* < 0.05, **: *p* < 0.01, ***: *p* < 0.001, ns: no significance.

## Abbreviations

The following abbreviations are used in this manuscript:

UC	Ulcerative Colitis
DSS	Dextran Sulfate Sodium
IBD	Inflammatory Bowel Disease
ZBTB	Zinc Finger and Broad-Complex, Tramtrack, and Bric-à-Brac
DAI	Disease Activity Index
CAC	Colitis-Associated Colorectal Cancer
