# Supplementary material for: ZBTB4 Deficiency Exacerbates DSS-Induced Colitis Through Activating NF-κB Pathway
Source: Cells. 2026 May 18;15(10):929. doi: 10.3390/cells15100929 (PMC13204054; doi:10.3390/cells15100929)
Supplement: Supplementary file 1 [file cells-15-00929-s001.zip › cells-4253513-supplementary.pdf]

Supplementary data

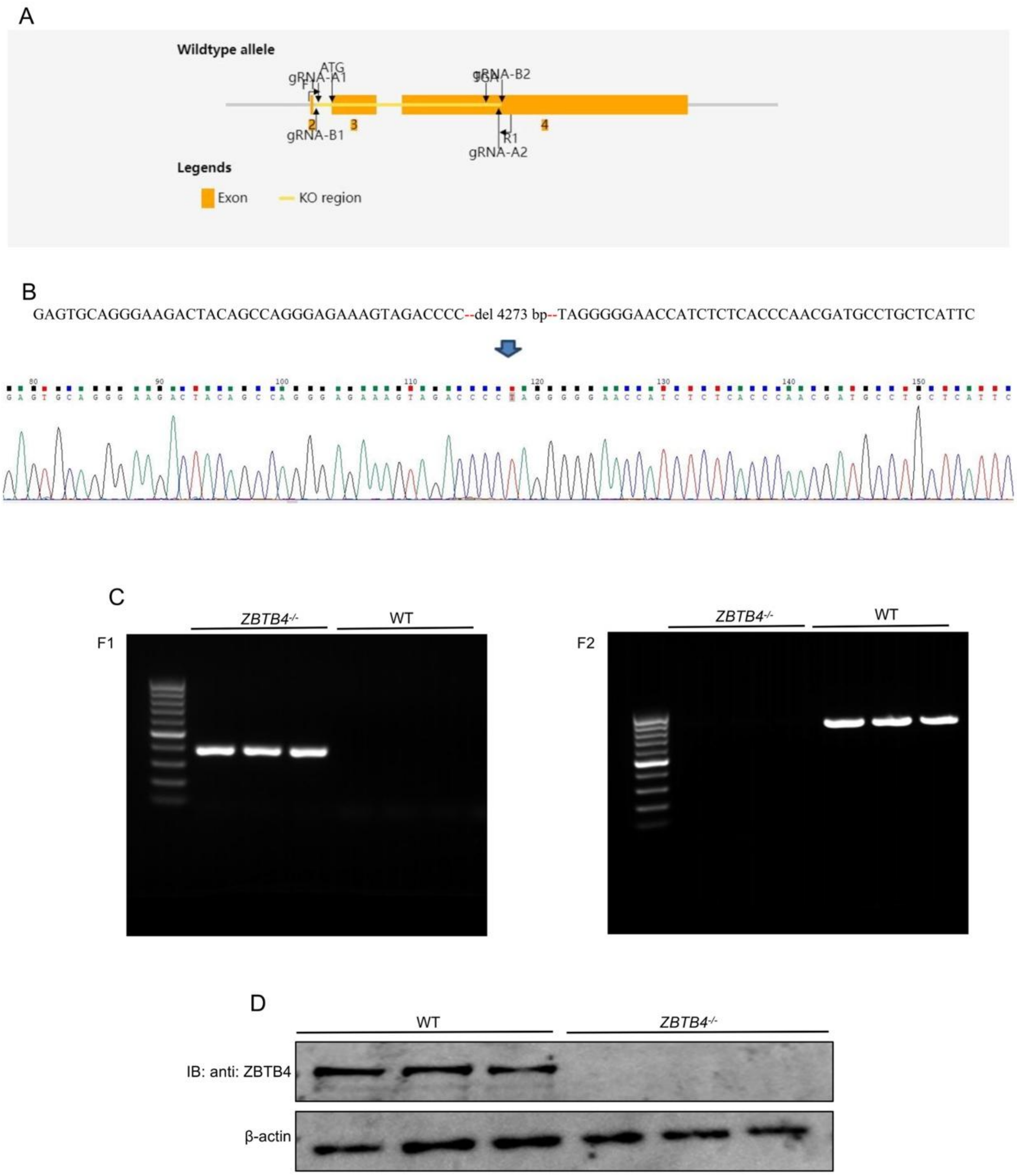

Figure S1. Construction strategy and identification of *ZBTB4*$^{-/-}$ mice. (A) Illustration of the *ZBTB4* knockout mouse generating protocol. (B) Seqencing validation of the Zbtb4 knockout allele. (C) Genotyping was performed using two primer pairs: F1/R1 and F2/R2. The F1/R1 produces a fragement only presnt in *ZBTB4*$^{-/-}$, whereas the F2/R2 produces a fragment only present in the wild-type allele. (D) The expression of ZBTB4 in WT mice and *ZBTB4*$^{-/-}$ mice were measured by Western Blot.

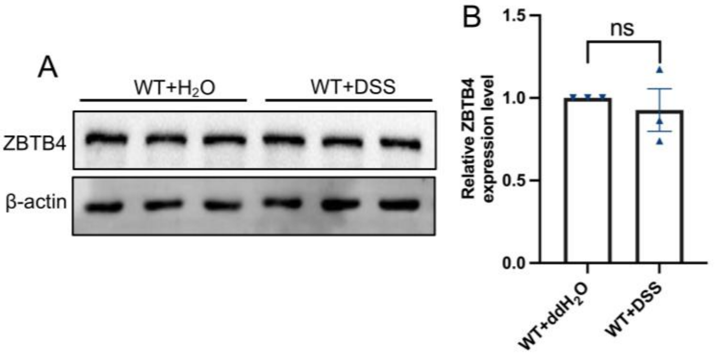

Figure S2. ZBTB4 expression in colon tissues from wild-type mice. (A-B) Protein levels of ZBTB4 in colonic tissues from WT mice with or without DSS-induced colitis were assessed by Western blot and quantitatively analyzed with three biological replicates for ZBTB4 expression by ImageJ software. Data are present as mean ± SEM.

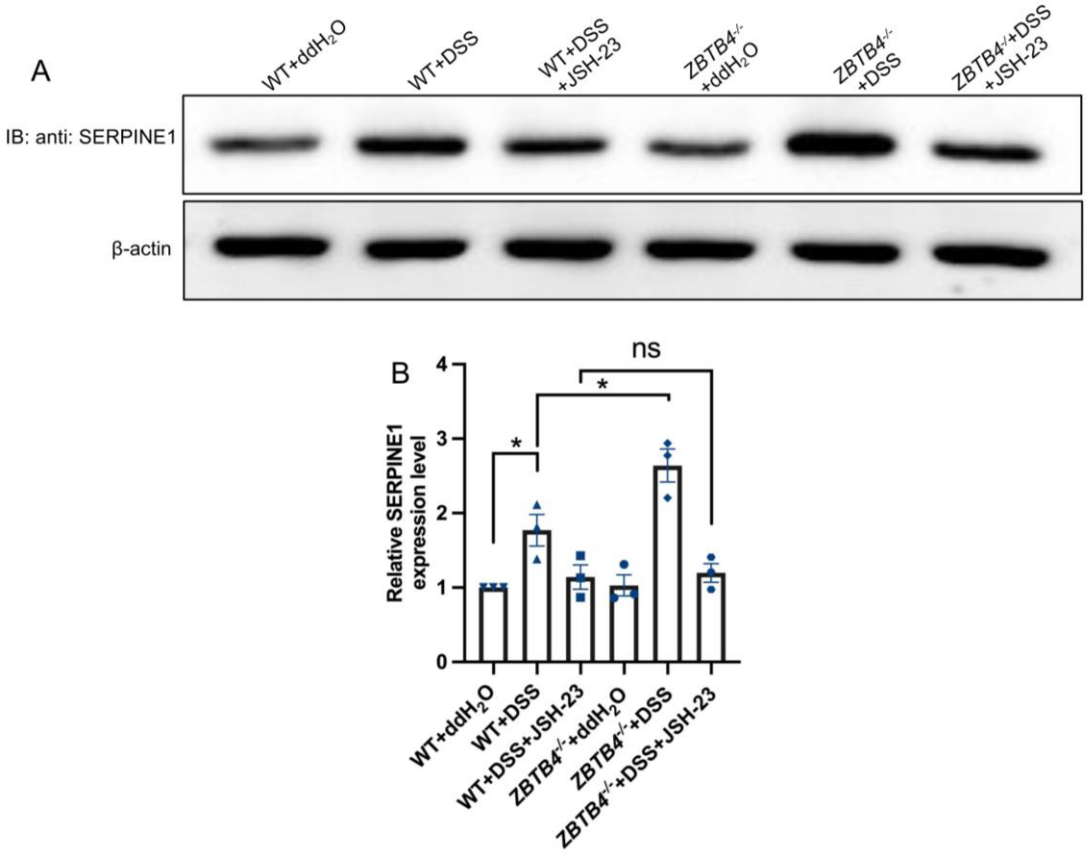

Figure S3. ZBTB4 deficiency increases colonic Serpine1 expression. (A-B) Protein levels of Serpine1 in colonic tissues from six groups of mice as indicated were assessed by Western blot and quantitatively analyzed with three biological replicates for Serpine1 expression by ImageJ software. Data are present as mean ± SEM. *: $p < 0.05$, ns: no significance.

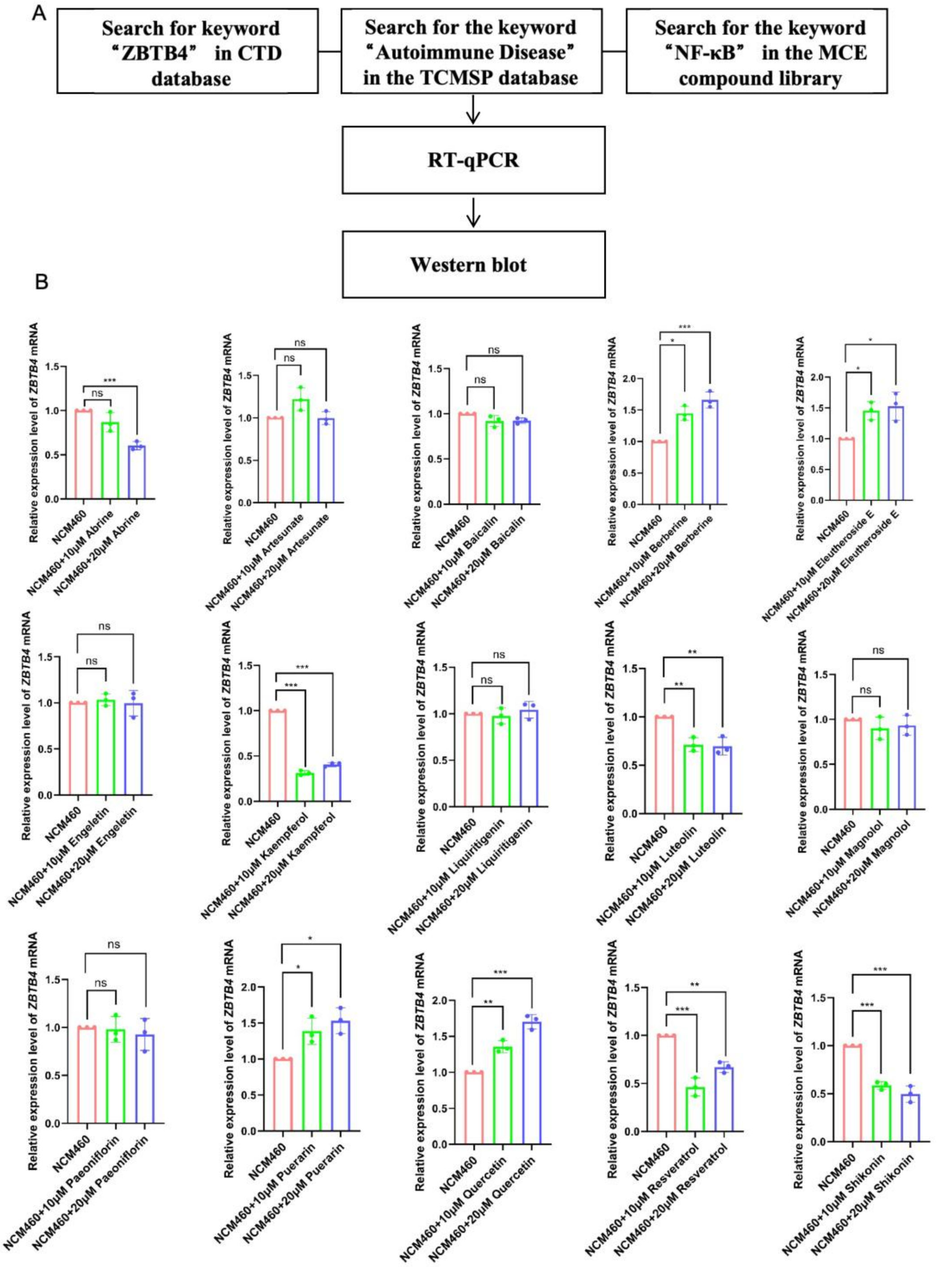

Figure S4. Candidate compounds screening in vitro. (A) The screen process of candidate compounds to regulate ZBTB4 expressions. (B) NCM460 cells were treated with compounds as indicated for 24 h. three independent experiments were conducted and data are present as mean ± SD. ZBTB4 mRNA levels were quantified by RT-qPCR. *: $p < 0.05$, **: $p < 0.01$, ***: $p < 0.001$, ns: no significance.
